# Impact of Simulation-Based Medical Education on Pre-clerkship Medical Students’ Confidence in Key Areas of Clinical Competence: An Exploratory Pre- and Post-survey Study

**DOI:** 10.7759/cureus.87059

**Published:** 2025-06-30

**Authors:** Danica K Friesen, Lyndon Rebello, Riley Reel, Victor Espinosa, Barbara Lelj Garolla Di Bard

**Affiliations:** 1 Faculty of Medicine, University of British Columbia, Vancouver, CAN; 2 Faculty of Medicine, Department of Emergency Medicine, University of British Columbia, Vancouver, CAN; 3 Research Informatics/Statistics, Vancouver Island Health Authority, Victoria, CAN; 4 Faculty of Medicine, Department of Pediatrics, University of British Columbia, Vancouver, CAN

**Keywords:** medical education, medical students, pre-clerkship, pre-clinical, simulation, simulation-based medical education

## Abstract

Background

Medical education is adapting to meet the growing demands of healthcare and patient care complexities. Traditional clinical training often relies on limited patient encounters, which may not fully develop clinical competence. Simulation-based medical education (SBME) offers controlled, immersive environments for practicing clinical skills and decision-making without risking patient safety. While SBME has been well-studied in advanced training, its effectiveness in first- and second-year medical students remains underexplored. This study aims to explore and quantify pre-clerkship medical students' perspectives on how SBME impacts confidence in clinical decision-making, communication, and clinical skills, compared to traditional learning methods alone.

Methods

This pre- and post-survey-based study assessed the impact of simulation (SIM) on students' self-reported confidence in clinical decision-making, communication, and clinical skills. Six simulation scenarios that aligned with the undergraduate medical curriculum of one Canadian institution were conducted from October 2023 to March 2024. Participants completed pre- and post-simulation surveys using 5-point Likert scales. A total of 67 surveys were analyzed.

Results

All 67 surveys were analyzed (35 pre-, 32 post-simulation) using one-sided Wilcoxon Signed Rank Tests. Pre-simulation responses indicated low baseline confidence, with only one item rated above neutrality. Post-simulation ratings showed statistically significant improvements across all domains (p < 0.01). Students also reported that they perceived simulation as more effective than traditional didactic learning in preparing them for clinical practice.

Conclusions

This exploratory study suggests that simulation-based education can enhance pre-clerkship students’ confidence in clinical decision-making, communication, and procedural skills, domains often underdeveloped at this stage of training. These findings offer early evidence that high-fidelity simulation may accelerate perceived clinical readiness. However, due to the small, self-selected sample, non-parallel survey design, and reliance on subjective outcomes, results should be treated as exploratory. Further multi-site studies using objective measures are needed to assess long-term impact on knowledge and skill retention as well as clinical performance.

## Introduction

Medical education continues to evolve to meet the demands of a rapidly changing healthcare landscape. The acquisition of clinical competence and proficiency in diagnostic reasoning is paramount for medical trainees in navigating the complexities of patient care and requires repetitive exposure to similar situations [1]. Traditionally, clinical education has relied on ad hoc encounters with patients to provide exposure to diverse presentations [1]. However, this approach often proves inefficient and inconsistent, failing to ensure timely and systematic exposure to an adequate quantity of clinical exposures for all learners and leaving competency up to chance [1-3]. Considering the high-risk nature of medical practice and its direct impact on patient health and well-being, this approach to medical training can be considered suboptimal [1]. Furthermore, medical education continues to face challenges, such as time constraints and limitations in clinical exposure, like those posed by the COVID-19 pandemic [4]. In light of this, simulation-based medical education (SBME) has emerged as a potential tool to mitigate disruptions in clinical training and to bridge the gap between didactic instruction and the complexities of clinical practice [2,4,5]. Simulation (SIM) in medical education refers to an artificial representation of a clinical scenario to achieve educational goals through experiential and reflective learning. Furthermore, “high-fidelity” simulation (HFS) refers to scenarios that are highly realistic and interactive for learners using mannequins or task trainers and are set in an environment that closely mimics an actual clinical setting [1]. 

SIM is being increasingly utilized in the pre-clerkship years in medical schools across North America, as indicated in the Association of American Medical Colleges (AAMC) 2011 survey on the use of SIM in medical education. Of the 90 participating medical schools across the United States and Canada, 86 used simulations in the preclinical years in areas such as “clinical skills, clinical medicine, and physical diagnosis” [6]. However, less than half of Canadian medical schools reported using simulation in pre-clerkship [6]. Currently, simulation is not being used extensively at the University of British Columbia (UBC) Medical School or in our distributed training program on Vancouver Island during the pre-clerkship years, and it is limited to two SIM scenarios immediately prior to clerkship.

A significant advantage of SIM is that it allows for the deliberate practice of a wide range of skills without risk to patients, which upholds the ethical imperative to “first do no harm” and provides an alternative to the traditional “see one, do one, teach one” approach [7-10]. Additionally, SIM provides an opportunity to practice non-technical skills, such as teamwork and crisis resource management. Incorporating simulations into preclinical education may provide a more holistic and experiential experience that supports the development of theoretical and clinical skills concurrently [2,9,11,12]. High-fidelity simulations have also been demonstrated to enhance long-term knowledge retention and improve the application of theoretical knowledge in clinical scenarios compared to traditional lectures [9,11,13,14]. While there is a large and growing body of evidence supporting the utility of simulation-based medical education in enhancing medical training, much of the existing research primarily focuses on experiences of trainees already exposed to clinical scenarios (i.e., third- and fourth-year medical students or residents). These studies consistently show that SIM is effective in improving technical skills, clinical decision-making, and teamwork [15,16]. However, there is limited research specifically examining the use of SIM to foster student preparedness before entering clinical environments in the third year.

In this vein, our study adds the voices of pre-clerkship medical students at the UBC distributed site on Vancouver Island to the discourse about integrating SIM earlier in medical education. Specifically, the aim of this study was to explore and quantify students' perspectives on the impact of simulation-based learning versus traditional didactic methods alone on improving confidence and perceived clinical preparedness in the areas of clinical decision-making, interdisciplinary communication, and performance of skills. In doing so, we seek to inform medical educators and curriculum developers about the role of simulation in medical education in pre-clinical years.

## Materials and methods

Study design and setting

The study employed a pre- and post-survey-based research design to evaluate the impact of simulation on the perceptions of learning and clinical preparedness among pre-clerkship medical students. We conducted six simulation scenarios that reflected concomitant classroom learning from the UBC Undergraduate Medical School curriculum. The simulations took place at the Island Health Centre for Interprofessional Clinical Simulation Learning (CICSL) high-fidelity simulation laboratories at the Royal Jubilee Hospital in Victoria, British Columbia. We administered pre- and post-simulation descriptive surveys to first- and second-year medical students using 5-point Likert scales to quantify their perspectives on the usefulness of simulation in developing their perceived clinical preparedness. Simulations were conducted from October 2023 to March 2024. Each session was 55 minutes and included a standardized pre-brief, a 15 to 20-minute simulation session, followed by a standardized debrief session.

Sample characteristics

The study population was first- and second-year medical students at UBC’s Island Medical Program in Victoria, British Columbia. Recruitment for the simulation sessions was conducted via social media using first and second-year UBC Island Medical Program private groups. Convenience sampling was incorporated, involving only students who attended a simulation session and who voluntarily completed the surveys. Surveys were anonymous and voluntary, and not completing them did not preclude students from participating in the simulation sessions. Based on the number of participants involved in the study, we opted to evaluate the first- and second-year cohorts as a single sample group for overall analysis.

Simulation development and administration

Each simulation scenario was aligned with weekly objectives from the UBC Undergraduate Medical School curriculum and was meant to solidify and expand on this learning by situating it in an experiential pseudo-clinical context. The scenarios were structured based on a simulation scenario template from the British Columbia Simulation Network (see Appendix). We aimed to conduct each simulation session within two to four weeks of the corresponding didactic material being learned in the classroom. One simulation for a chief complaint of chest pain was administered four weeks after content learning due to inclement weather, necessitating rescheduling. All other simulations were administered within two weeks of classroom learning. Year one and two medical students had separate simulation scenarios based on their academic curriculum. Each scenario was peer-reviewed by a member of the research team, a first-year Emergency Medicine resident, to ensure high quality and an appropriate level of complexity for first- and second-year medical students. This was to mitigate reports in the literature of simulation experiences being overwhelming when simulation scenarios and expectations are significantly mismatched to the level of training, which can impair the ability to meet the educational objectives of the simulation [11]. 

We invited local junior residents in Emergency Medicine, Family Medicine, Anesthesia, and Internal Medicine programs to facilitate the simulations, with a total of seven different residents participating. Residents were sought as teachers in light of the “near-peer” effect, which has been documented to have benefits for healthcare student learning, including improved self-efficacy in psychomotor skills, interprofessional skills, and critical thinking [17]. Multiple online and in-person sessions of the CICSL Simulation Facilitation Workshop were offered to all participating residents. Three out of seven residents obtained the certificate. We endeavored to standardize the simulation and pre- and post-debriefing process by providing all residents with instructions for the simulation timeframe as well as a pre-briefing checklist from BC Children’s and Women’s Hospital, as well as the PEARLS debriefing tool [18,19]. These resources were part of the CICSL Simulation Facilitator training course and were also endorsed by the British Columbia Simulation Network. 

Simulation environment and equipment

The CICSL simulation laboratories replicated an operating room and an emergency department trauma bay, designed to enable students to apply relevant skills under pressure in an out-of-classroom simulated environment. The mannequins used (Laerdal© SimMan 3G) were controlled remotely by two second-year medical students who had completed the CICSL Simulator Operations Workshop. These mannequins can display a range of physiological and neurological symptoms as well as pharmacological responses for over 145 drugs. Learners could visualize relevant assessment findings, like central cyanosis or pupillary changes, and auscultate normal and abnormal heart, lung, and abdominal sounds. They could also perform multiple procedures on the mannequins, including inserting intravenous lines and endotracheal intubation. A separate conference room was available for pre-brief and debrief.

Survey design

The pre-survey consisted of seven items divided into three sections (Table 1), and the post-survey comprised ten items divided into four sections (Table 2). These surveys were designed to quantify the impact simulation had on three outcome measures related to students’ perceived confidence and preparedness for clinical practice: (1) confidence in clinical decision making, (2) skills in interdisciplinary communication, and (3) confidence in performing relevant clinical skills.

**Table 1:** Pre-survey questions

Question Number	Clinical Aspect	Question Text
Q1.1	Confidence in clinical decision making	I am prepared to respond to changes in my patient’s condition
Q1.2	Confidence in clinical decision making	I am confident in my ability to prioritize care and interventions
Q2.1	Skills in interdisciplinary communication	I have an understanding of members of the healthcare team I can consult for help
Q2.2	Skills in interdisciplinary communication	I am confident in my ability to share ideas and keep team members informed using closed-loop communication
Q2.3	Skills in interdisciplinary communication	I am confident in my ability to use structured communication techniques (e.g., SBAR) during handover
Q3.1	Confidence in performing relevant clinical skills	I am confident in my physical assessment skills
Q3.2	Confidence in performing relevant clinical skills	I have a good understanding of medications pertinent to the treatment of this condition

**Table 2:** Post-survey questions Italicized questions indicate the three questions unique to the post-survey.

Question Number	Clinical Aspect	Question Text
Q1.1	Confidence in clinical decision making	I had the opportunity to practice my clinical decision-making skills
Q1.2	Confidence in clinical decision making	I am better prepared to respond to changes in my patient's condition
Q1.3	Confidence in clinical decision making	I am more confident in my ability to prioritize care and interventions
Q1.4	Confidence in clinical decision making	Debriefing was valuable in helping me improve my clinical judgment
Q2.1	Skills in interdisciplinary communication	I developed a better understanding of members of the healthcare team I can consult for help
Q2.2	Skills in interdisciplinary communication	I am more confident in my ability to share ideas and keep team members informed using closed-loop communication
Q2.3	Skills in interdisciplinary communication	I am more confident in my ability to use structured communication techniques (e.g., SBAR) during handover
Q3.1	Confidence in performing relevant clinical skills	I am more confident in my physical assessment skills.
Q3.2	Confidence in performing relevant clinical skills	I developed a better understanding of medications used to treat this condition (select N/A if no medications in this scenario)
Q4.1	Comparison to traditional didactic learning	Do you agree/disagree that simulation is useful in consolidating knowledge and preparing you for clinical practice as compared to traditional didactic lectures and case-based learning alone

Questions for the sections evaluating the above three outcome measures were adapted from the multi-study validated Simulation Effectiveness Tool-Modified (SET-M) with permission [20]. Each question required a selection on a 5-point Likert scale (strongly disagree/disagree/neutral/agree/strongly agree) and had an option to select “not applicable” to protect against arbitrary responses. While both surveys drew from validated SET-M survey items, the post-survey included three additional items that could only be answered following the simulation experience. These items assessed whether students had the opportunity to practice decision-making, the value of debriefing for their clinical reasoning, and the perceived usefulness of simulation compared to didactic learning. These differences in item structure introduced limitations in directly comparing pre- and post-survey responses on a one-to-one basis. 

Survey administration

Participants accessed the surveys on their mobile devices after scanning printed QR codes only available at the simulation event. Each participant filled in a forced-entry free-text code at the beginning of each survey that consisted of the first three letters of their mother’s maiden name and their personal birth month as a number (e.g., WON04 for Wong, April). This prevented “multiple entry” of a single participant and ensured complete anonymity of survey data collected. Ease of completion via mobile-friendly software and an estimated survey completion time of five minutes attempted to minimize non-response bias. Each Likert scale question in the surveys was a forced response question, which aimed to prevent incomplete survey results from being included in the study. However, since survey participation was completely voluntary, we did not always have engagement by all students or equal numbers of pre- and post-surveys completed for each simulation. 

Data collection

Neither the research team nor the simulation facilitators were privy to whether surveys had been completed prior to participating in the simulation session. The total number of pre- and post-surveys completed was tallied, resulting in a final sample size of n = 67. The data were collected between October 2023 and March 2024.

Statistical analysis

Although both pre- and post-simulation surveys were administered, the data were not analyzed using matched-pairs statistical methods. This decision was based on the differing constructs measured by the survey items: pre-simulation questions assessed baseline confidence or competence, whereas post-simulation questions asked participants to rate their improvement relative to their pre-simulation state. Because these items were not directly comparable, matched analyses such as the Wilcoxon Signed Rank test for paired data were deemed inappropriate. Furthermore, the post-survey included questions that had no pre-survey equivalent, making direct item-level comparisons inappropriate. 

While 60 of the 67 total responses could be matched by participant and simulation type, a Mann-Whitney U test was also not used due to the violation of the assumption of independence between groups. Using this test under such conditions would compromise the validity of the resulting p-values and reduce the statistical power, thereby limiting the strength of any inferences.

Instead, we conducted one-sample Wilcoxon Signed Rank tests separately for each survey item at both time points. These tests assessed whether the median response differed significantly from the neutral midpoint of 3 on the 5-point Likert scale. One-sided tests of superiority (H₁: median > 3) were used, as the primary interest was in detecting potential benefits of the simulation intervention. The possibility of negative effects was considered unlikely in this educational context and was therefore not tested, allowing for greater statistical power to detect positive effects. 

We opted for a more stringent significance level (p < 0.01) instead of the conventional p < 0.05 to mitigate the risk of Type I error, particularly in light of our modest sample size and the exploratory nature of the study. Smaller samples are associated with reduced statistical power, which can increase the likelihood of incorrectly identifying an effect as significant. By selecting a lower p-value threshold, we aimed to strengthen the robustness and reliability of any detected effects. We recommend that future studies with larger sample sizes further validate these findings.

Surveys were designed using forced-response fields, which should have prevented submission with unanswered items. Despite this, a small proportion of missing data (0.5%) was identified. While the cause could not be definitively verified, it may reflect a rare technical issue within the Qualtrics platform or user-side interruptions (e.g., browser errors or connectivity problems). Given the very low frequency and random distribution of these instances, we treated the missing responses as missing at random (MAR) and conducted all analyses based on the number of valid responses available per item. Importantly, these analyses were not designed to track within-subject change. Rather, the focus was on group-level perceptions of benefit from participation in the simulation-based intervention. All statistical analyses were performed using the Statistical Package for the Social Sciences (SPSS) software, version 25 (IBM Corp., Armonk, NY).

Ethics statement

This study received ethics approval under UBC’s Behavioral Research Ethics Board Class Project Certificate H16-00044. The work was carried out in accordance with the Declaration of Helsinki, including, but not limited to, the guarantee of participant anonymity and the obtaining of informed consent.

## Results

In total, six simulation sessions were completed. Sessions ranged from four to 10 participants, totaling 41 participants across all six sessions. Some students attended more than one simulation. We collected a total of 35 pre-surveys and 32 post-surveys, for a total of 67 discrete survey responses (Table 3).

**Table 3:** Description of Simulation Scenario, Content, Audience, and Number of Pre and Post Surveys Completed ^a^ For this simulation, students were told the diagnosis (anaphylaxis) from the outset; ^b^ Simulation provided four weeks after classroom learning due to inclement weather necessitating rescheduling;​​​​​​​ ^c^ Y1 = year one medical students, Y2 = year two medical students.

Simulation Scenario	Associated Curriculum Content	Audience	Number of Simulation Participants	Pre-survey	Post-survey	Total surveys
Rash^a^	Immunology, allergy, anaphylaxis	Y1^c^	10	5	6	11
Dyspnea	Venous thromboembolism, pulmonary embolism	Y2^c^	5	5	6	11
New Weakness	Neuroanatomy of cortex, stroke	Y1	5	4	4	8
Chest Pain^b^	Atherosclerosis, angina, myocardial infarction	Y2	8	8	5	13
Headache	CNS infections, meningitis, encephalitis	Y1	9	9	8	17
Abdominal Pain	Sepsis, biliary tree disease	Y2	4	4	3	7
Totals	-	-	41	35	32	67

Pre-simulation student perspectives on preparedness for clinical practice

**Figure 1:**
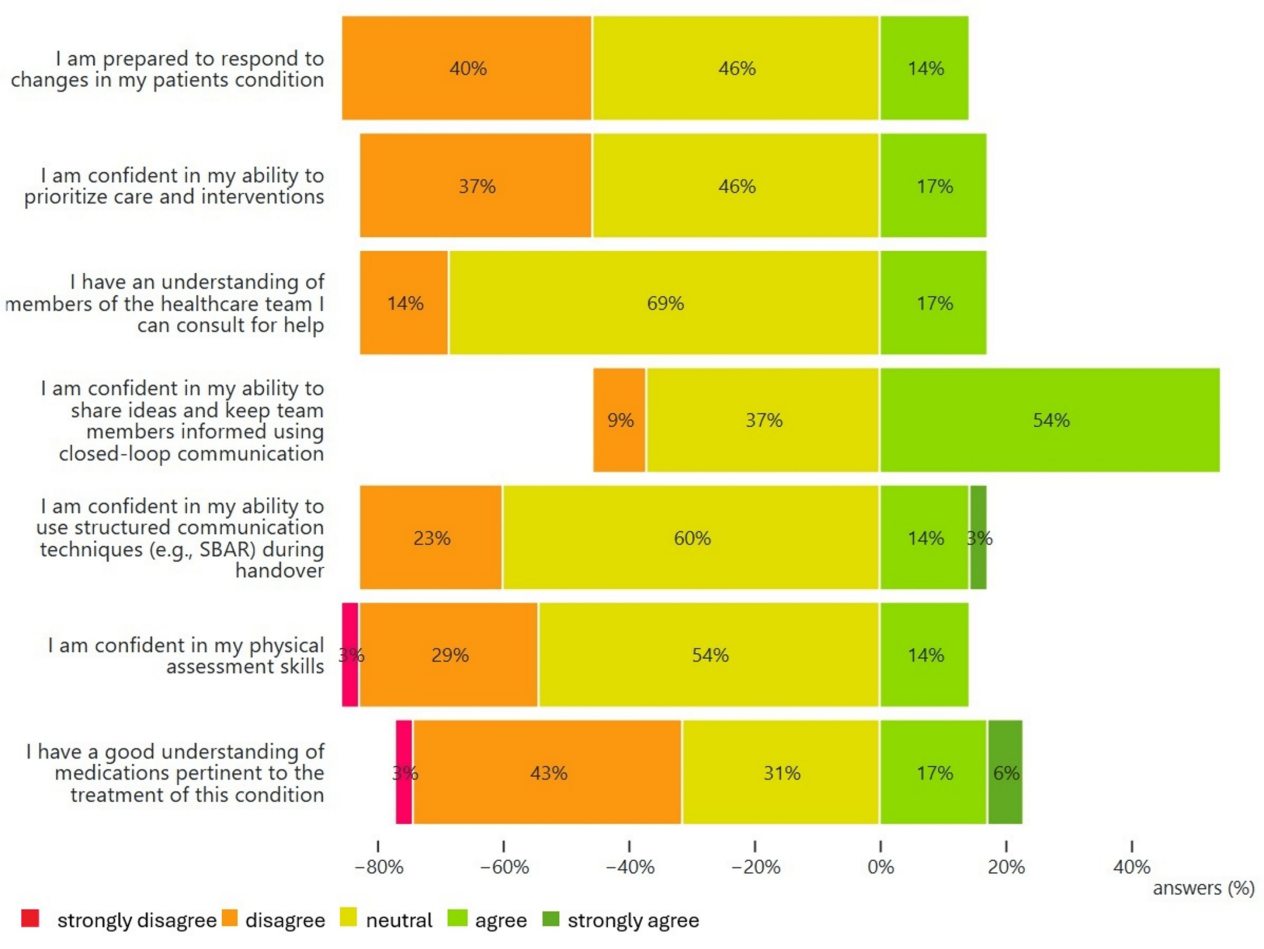
Student ratings of outcome measures related to clinical preparedness prior to simulation (n=35)

We performed a one-sample one-sided Wilcoxon Signed Rank Test on all pre-simulation surveys (n=35) (Figure 1). The test was conducted to compare superiority to a median value of 3, which corresponded to “neutral” on the Likert scale. For all but one question, responses did not show a median superior to 3, with a 99% confidence (p<0.01) (see Table 4). This meant that participants did not feel confident or prepared regarding those statements or factors prior to the simulation. The exception was “I am confident in my ability to share ideas and keep team members informed using closed-loop communication,” where about half of the participants agreed and the other half disagreed or felt neutral.

**Table 4:** Summary of pre-simulation responses: median, minimum, and maximum Likert scores

	N	Median	Minimum	Maximum
I am prepared to respond to changes in my patient's condition	35	3	2	4
I am confident in my ability to prioritize care and interventions	35	3	2	4
I have an understanding of members of the healthcare team I can consult for help	35	3	2	4
I am confident in my ability to share ideas and keep team members informed using closed-loop communication	35	4	2	4
I am confident in my ability to use structured communication techniques (e.g., SBAR) during handover	35	3	2	5
I am confident in my physical assessment skills	35	3	1	4
I have a good understanding of medications pertinent to the treatment of this condition	35	3	1	5

Post-simulation student perspectives on preparedness for clinical practice

**Figure 2:**
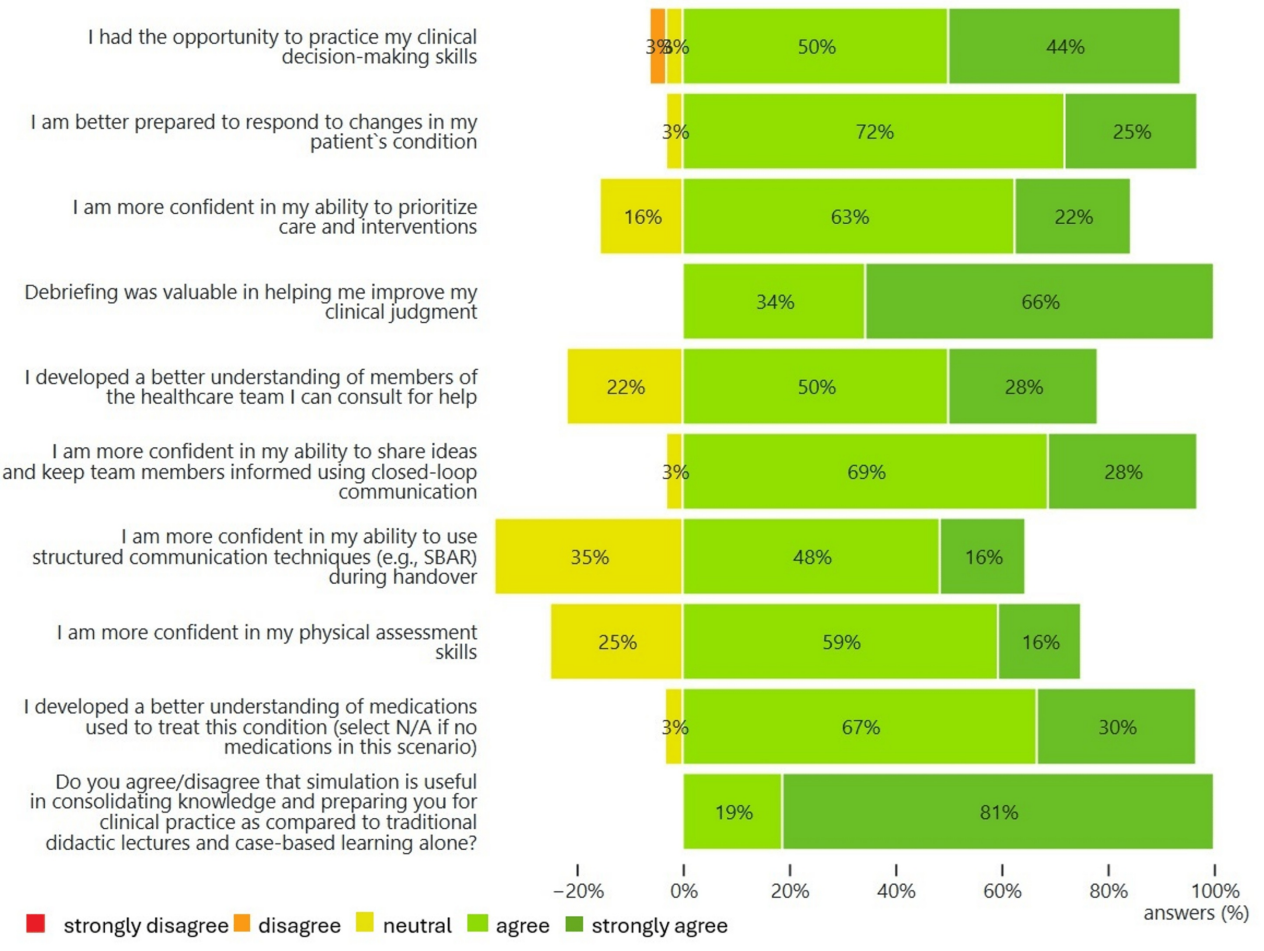
Student ratings of outcome measures related to clinical preparedness after simulation (n=32)

We then performed the same one-sample one-sided Wilcoxon Signed Rank Tests on all post-simulation surveys (n=32) (Figure 2). The test was conducted to compare superiority to a median value of 3, which was equivalent to “neutral” on the Likert scale. For all questions, responses show a median superior to 3, with a 99% confidence (p<0.01) (see Table 5). This meant that participants felt more confident or prepared after the simulation with respect to those statements or factors before the simulation.

**Table 5:** Summary of post-simulation responses: median, minimum, and maximum Likert scores

	N	Median	Minimum	Maximum
I had the opportunity to practice my clinical decision-making skills	32	4	2	5
I am better prepared to respond to changes in my patient's condition	32	4	3	5
I am more confident in my ability to prioritize care and interventions	32	4	3	5
Debriefing was valuable in helping me improve my clinical judgment	32	5	4	5
I developed a better understanding of members of the healthcare team I can consult for help	32	4	3	5
I am more confident in my ability to share ideas and keep team members informed using closed-loop communication	32	4	3	5
I am more confident in my ability to use structured communication techniques (e.g., SBAR) during handover	31	4	3	5
I am more confident in my physical assessment skills	32	4	3	5
I developed a better understanding of medications used to treat this condition (select N/A if no medications in this scenario)	30	4	3	5
Do you agree/disagree that simulation is useful in consolidating knowledge and preparing you for clinical practice as compared to traditional didactic lectures and case-based learning alone?	32	5	4	5

## Discussion

Positioning our findings within the simulation literature

A Unique Early-Stage Lens

Most simulation-based medical education (SBME) research has centered on clerkship or postgraduate learners who are already immersed in clinical environments. Our study deliberately targets pre-clerkship students-learners who have minimal patient contact and whose clinical identity is still forming. By doing so, we provide the first cross-sectional, pre- and post-survey evidence that suggests SBME can accelerate perceived readiness before students ever set foot on the wards. This early-stage focus distinguishes our work from prior studies that used simulation mainly to reinforce basic-science concepts or to teach isolated procedural skills to pre-clerkship cohorts [21-23].

Expanding the Outcome Palette

Earlier pre-clerkship studies reported gains in understanding basic science [21,22] or reductions in procedural anxiety [3,23]. We extend the conversation by quantifying confidence in two domains that had not been examined at this level of training: clinical decision-making and closed-loop communication. We also added support for how SBME can improve confidence in clinical skills, which is in line with previous studies [22]. These domains map directly onto the CanMEDS roles students will soon be assessed on, giving program directors concrete signals that SBME can nurture competence milestones earlier than traditional lectures alone.

Magnitude of Effect

Across all three domains, confidence improved significantly after just a single simulation exposure, with baseline scores largely negative or neutral. These baseline ratings underscore how little opportunity pre-clerkship students have to practice clinical decision-making, team communication, and clinical skills in didactics; the robust post-simulation gain suggests that SBME can fill this gap.

Why “Soft” Matters

Although self-reported confidence is a subjective metric, it is tightly linked to student engagement, satisfaction, and academic achievement [24-26]. In the pre-clerkship context, where anxiety and impostor feelings run high-bolstering confidence can free cognitive bandwidth for knowledge application and patient-centered behaviors. Our data, therefore, add to the argument that simulation is not merely a “nice to have” enrichment, but a strategic lever for pre-clerkship curriculum quality. That said, confidence is not equivalent to competence. While heightened self-efficacy may support engagement and motivation, our findings should not be interpreted as definitive evidence that SBME improves objective clinical performance. Future research must incorporate validated performance-based outcomes to examine whether perceived confidence translates to skill acquisition or improved patient care.

Limitations

This study has several important limitations that should be considered when interpreting the findings. First, the voluntary nature of participation and recruitment through peer social media channels introduces a risk of self-selection bias. Students with a pre-existing interest in simulation or experiential learning may have been more inclined to attend and complete surveys, potentially inflating positive outcomes. However, this type of engagement is representative of the real-world population most likely to seek out or benefit from early SBME opportunities, and understanding their perceptions remains valuable for curriculum planning.

Second, although the study employed a pre/post design, the surveys were not fully parallel: the post-survey included three items that were not assessable prior to simulation. This structural discrepancy limits direct item-to-item comparisons. Nonetheless, the majority of items did align across both surveys and still permitted meaningful directional analysis of change in perceived confidence. The paired pre/post design still offers a more robust estimate of perceived change over time than single-time-point surveys.

Third, although the surveys were designed with forced-response fields, a small number of responses (0.5%) were missing. While the cause could not be definitively determined, these were attributed to potential technical issues such as browser interruptions or survey platform anomalies. The minimal proportion of missing data and the lack of a pattern across items suggest a limited risk of bias.

Fourth, the observed gains in confidence cannot be definitively attributed solely to the simulation intervention. Factors such as concurrent classroom learning (natural progression) or the Hawthorne effect may have influenced students’ perceptions of improvement. Although confidence changes cannot be definitively attributed to simulation alone, the consistent positive direction of post-simulation responses across domains provides useful exploratory insight. These findings can help guide the generation of hypotheses and the design of future studies using objective outcome measures and controlled conditions.

Fifth, variability in facilitator experience may have affected the consistency of simulation delivery. Only three of the seven resident facilitators had completed formal simulation facilitation training. Despite this, all facilitators received standardized orientation materials and debriefing frameworks and were selected for their near-peer relevance, which has been shown to enhance student engagement and reliability. Additionally, the amalgamation of first- and second-year students into a single analytic cohort may have introduced confounding, although both groups share similarly limited clinical exposure, and each simulation was matched to year-specific curricular content. The study was also conducted at a single site, the University of British Columbia’s Island Medical Program, which may limit external validity. Differences in institutional culture, simulation infrastructure, curricular sequencing, and learner demographics may reduce the applicability of findings to other medical schools.

Taken together, these limitations constrain both the internal and external validity of the study. Although statistical significance was observed across all items, the absence of a control group, lack of objective performance metrics, non-parallel survey design, and other methodological constraints limit the strength of causal inferences. These results should therefore be interpreted as exploratory. Future research should aim to replicate these findings in larger, multi-center studies using standardized facilitator training, validated parallel survey instruments, and objective competency assessments.

Implications for undergraduate medical education

These findings offer preliminary insight into how simulation-based medical education may enhance self-perceived confidence among pre-clerkship students in core domains such as clinical decision-making, communication, and procedural skills. While confidence is not a proxy for competence, early gains in self-efficacy can contribute to learner engagement, reduce anxiety, and foster readiness for clinical environments, particularly in students with limited patient exposure. The use of high-fidelity simulation aligned with curriculum content provides a promising model for bridging the gap between didactic learning and clinical application. Although several limitations constrain generalizability, this study highlights the potential utility of structured simulation in the pre-clinical years as a complement to traditional instructional methods. Curriculum designers may consider these early findings when developing programs to support foundational clinical skills and learner confidence.

## Conclusions

In conclusion, this exploratory study suggests that simulation-based education may positively influence pre-clerkship students' confidence in clinical decision-making, communication, and procedural tasks. These findings reflect early perceptions of benefit and support the role of simulation as a potentially valuable adjunct to traditional didactic methods. However, due to the limitations outlined, including a small, self-selected sample, non-parallel survey tools, and reliance on subjective outcomes, these results should be interpreted judiciously. Further controlled, multi-institutional studies incorporating objective measures of competence are needed to evaluate whether such confidence gains translate into clinical preparedness and improved performance.
